# Recent advances of on-demand dissolution of hydrogel dressings

**DOI:** 10.1186/s41038-018-0138-8

**Published:** 2018-12-29

**Authors:** Hao Lu, Long Yuan, Xunzhou Yu, Chengzhou Wu, Danfeng He, Jun Deng

**Affiliations:** 1 Department of Dermatology, Chongqing Traditional Chinese Medicine Hospital, Chongqing, 400021 China; 2 Institute of Burn Research, South-West Hospital, State Key Lab of Trauma, Burn and Combined Injury, Third Military Medical University (Army Medical University), Gaotanyan Road No. 30, Shapingba District, Chongqing, 400038 China; 3 Department of Breast Surgery, Southwest Hospital, Third Military Medical University (Army Medial University), Chongqing, 400038 China; 4 Department of Respiratory, Wuxi Country People’s Hospital, Chongqing, 405800 China

**Keywords:** Wound management, Wound dressing, On-demand dissolution, Hydrogel

## Abstract

Wound management is a major global challenge and a big financial burden to the healthcare system due to the rapid growth of chronic diseases including the diabetes, obesity, and aging population. Modern solutions to wound management include hydrogels that dissolve on demand, and the development of such hydrogels is of keen research interest. The formation and subsequent on-demand dissolution of hydrogels is of keen interest to scientists and clinicians. These hydrogels have excellent properties such as tissue adhesion, swelling, and water absorption. In addition, these hydrogels have a distinctive capacity to form in situ and dissolve on-demand via physical or chemical reactions. Some of these hydrogels have been successfully used as a dressing to reduce bleeding in hepatic and aortal models, and the hydrogels remove easily afterwards. However, there is an extremely wide array of different ways to synthesize these hydrogels. Therefore, we summarize here the recent advances of hydrogels that dissolve on demand, covering both chemical cross-linking cases and physical cross-linking cases. We believe that continuous exploration of dissolution strategies will uncover new mechanisms of dissolution and extend the range of applications for hydrogel dressings.

## Background

Wound healing is intrinsically and closely related to survival; wounds such as the diabetic foot ulcers that fail to heal can lead to a lower 5-year survival rate than some cancers (e.g., breast and prostate) [1, 2]. Thus, many efforts have been devoted to developing new and effective strategies to promote wound healing. In clinical settings, the standard processes for wound treatment include as follows: (1) cleaning the wound, (2) debriding, (3) choosing suitable dressings for wound healing, and(4) binding the wound to avoid shedding of dressings [3]. Dressings have long been considered as a critical part of wound care, and many of dressings can indeed be useful for topical therapies. In addition to the traditional cotton gauze dressings, a myriad of new dressings that are made of biological materials can also be selected by the clinicians. Over the past few decades, biomaterials and especially polymeric materials have rapidly become a key enabling technology in this push to develop advanced strategies for wound care [4–6]. Polymeric materials were mostly used as dressing to treat wounds, which can absorb wound exudates, prevent wound desiccation, and isolate the wound from the environment [6–8]. A range of commercially available polymer-based dressings, such as hydrocolloids, polymeric film, fibers, and hydrogels, have been widely used for the treatment [5, 9]. However, currently available dressings adhere to the wound, particularly burn wound surfaces, requiring cutting and mechanical debridement for a dressing change. This can lead to traumatization of newly epithelialized tissues, delayed healing, and personal suffering in the injured patient [10, 11]. Additionally, changing a wound dressing takes a long time. For example, the average duration of a burn dressing change is almost an hour [10]. The dressing change for anesthesia can require even more time. More seriously, these painful dressing changes have to be conducted many times until an obvious improvement in the wound healing is observed. This means that opaque burn dressings in clinical applications should be changed every 2 days to observe the condition of wound and avoid excessive waste of dressings. In fact, doctors want to use transparent dressings to make it easier to monitor the condition of wound and to place it on the wound surface for a long time until it heals. If the wound is infected, the dressings are expected to be dissolved as soon as possible. The ideal dissolution time of dressings should be controlled within a few minutes, and the ideal time for dressing change is expected to be rapid. Consequently, strategies with gentler and less invasive approaches enable facile dressing change and avoid secondary damage.

Therefore, there is a critical unmet need for a topically applied material that (1) is easily applied and forms in situ, (2) is of sufficient mechanical flexibility to accommodate complex wound contours and volumes, (3) can be easily and atraumatically removed under controlled conditions for definitive surgical care, and (4) is non-toxic. In order to fulfill these requirements, the first dissolvable hydrogel dressings were introduced. These controlled dissolution of hydrogels is especially significant for (1) atraumatic removal after dressing function is completed, (2) targetable transit of sealed therapeutics (e.g., proteins, cells, and small molecules), and (3) customized administration of highly efficient agent [12]. Despite an intense research focus on dissolvable cross-linked hydrogel, little effort has been made to summarize these systems. Here, we will outline the recent advances in hydrogels that dissolve on demand for nursing wounds, as this rapidly evolving field continues to make important contributions to biomedicine.

## Review

### Dissolvable cross-linked hydrogels

**Fig. 1:**
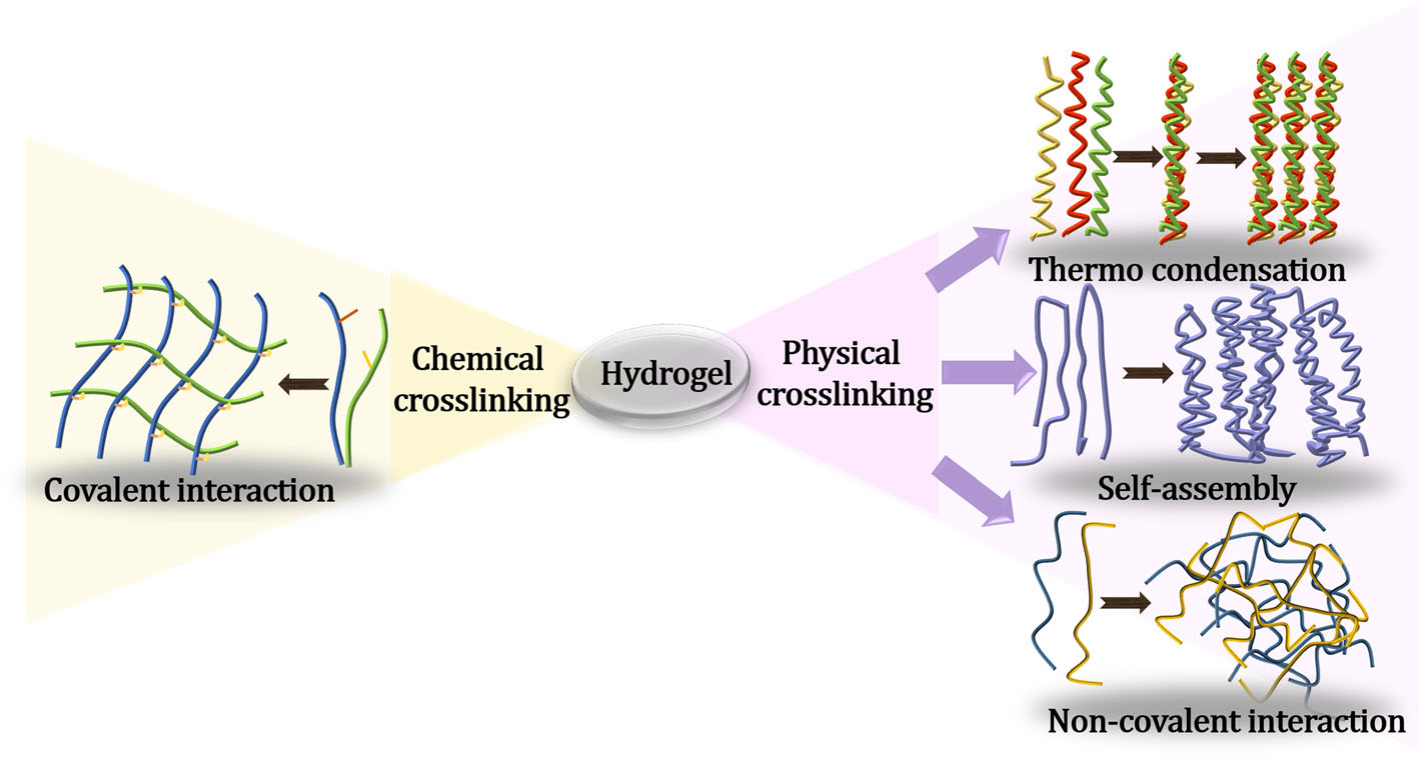
Schematic illustration of hydrogels fabricated through chemically cross-linked or physically cross-linked

Hydrogels have three-dimensional structure, cross-linked networks, and excellent hydrophilicity [13]. Generally, hydrogels can be classified as either physically or chemically cross-linked hydrogels (Fig. 1). The network of physically cross-linked hydrogels is formed via non-covalent forces, such as physically molecular entanglements, ionic forces, host-guest interactions, H-bonding, and hydrophobic forces [14]. Because their networks are formed and decomposed by stimuli, such as changes of pH, ionic concentration, or temperature, physically cross-linked hydrogels can exhibit reversibility [15]. On the other hand, the networks of chemically cross-linked hydrogels are formed through covalent bonds [16]. These types of hydrogels can only be dissolved by adding a dissolving agent along with a chemical reaction. Compositionally, both natural polymers including commonly used collagen, hyaluronic acid (HA) and chitosan (CS), and synthetic polymers involving poly(vinyl alcohol), poly(ethylene glycol) (PEG), and poly(acrylic acid) are widely utilized to form the hydrogels [17–19].

In recent years, hydrogels have become attractive for wound healing applications owing to their biocompatibility, tunable biodegradability, and controllable mechanical properties [20–22]. Since the chemistries and performances of hydrogels are controllable and repeatable, synthetic hydrogels gain especially interests for wound care applications [23, 24]. These controllable features include mediating the hydrogel and dissolution rates [25, 26]. In particular, the controlled dissolution of hydrogels is important for their atraumatic removal from the wound after their function is complete. Generally, the dissolution of chemically cross-linked hydrogels can be achieved by incorporating cleavable moieties though the approach of ester hydrolysis or enzymatic degradation [27, 28]. Compared with chemically cross-linked hydrogels, the physically cross-linked hydrogels can undergo phase transitions by changing the external environment without coupling agent, optical irradiation, or organic solvents (which are usually harmful to the human body) [29–31]. However, mechanical properties of physically cross-linked hydrogels are weaker than chemically cross-linked hydrogels, so their application is limited [32, 33]. Consequently, we will review recent advances in developing dissolvable physically and chemically cross-linked hydrogels.

#### Chemically cross-linked hydrogels

Thiol-thioester exchange reaction, thiol-disulfide exchange reaction, retro-Michael reaction, and retro Diels-Alder (rDA) reaction have all been used to prepare dissolvable hydrogels. These hydrogels offer responsive synthetic processing for dissolution rates. Examples of these reactions as a method to synthesize on-demand dissolvable hydrogels are described below.

##### Thiol-thioester exchange

**Fig. 2:**
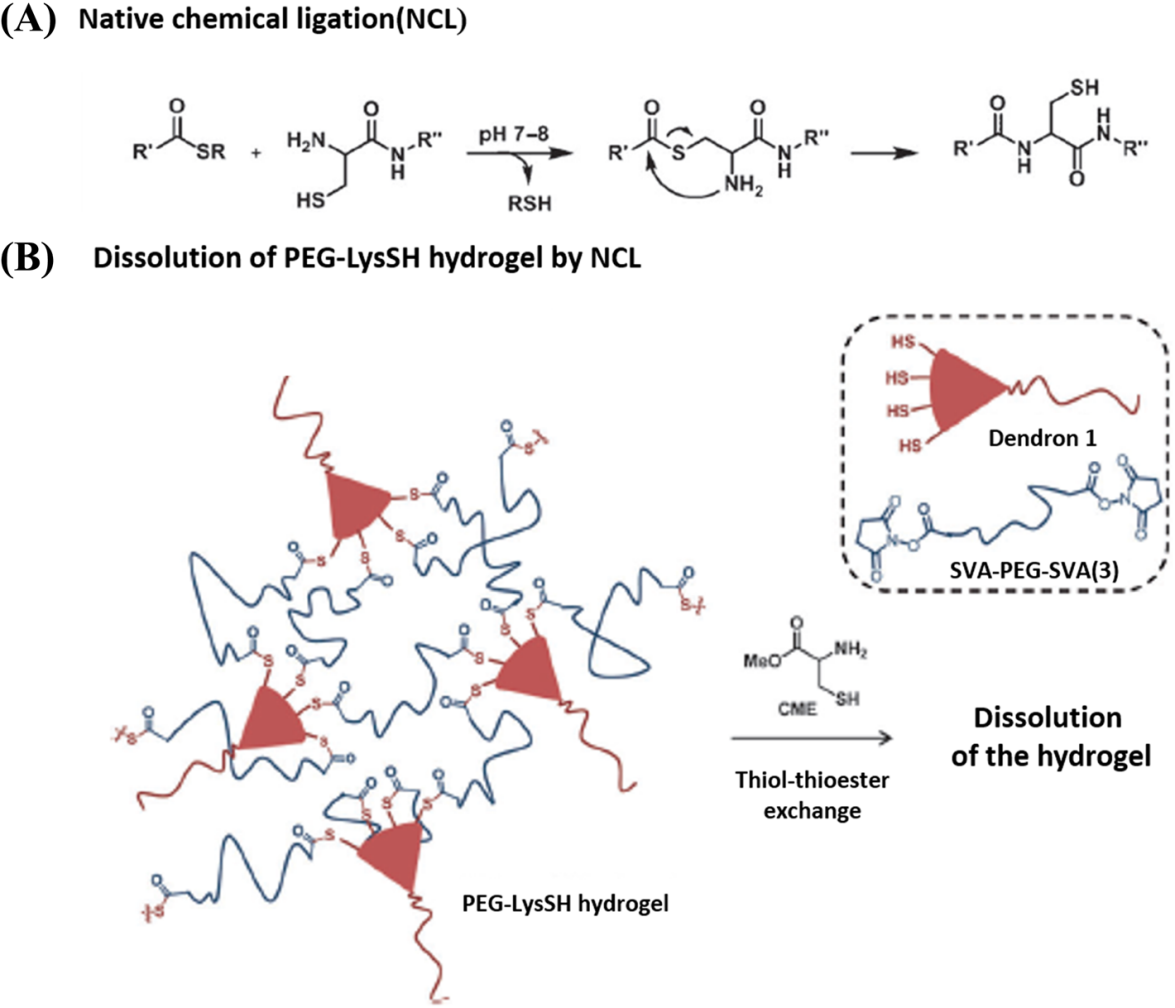
Formation of poly(ethylene glycol) lysine sulfhydryl (PEG-lysSH) and subsequent dissolution. **a** Thiol-thioester exchange reaction [37]. **b** The example of a hydrophilic PEG-lysSH hydrogel dissolution based on thiol-thioester exchange. Figures are adapted with permission from the original articles of Ghobril et al. [37] (Copyright 2013 by Wiley-VCH Verlag GmbH & Co. KGaA, Weinheim). *CME* cysteine methyl eater

Thiol-thioester exchange reaction can happen in water within biological pH ranges, which is mostly applied in self-assembly purposes where physiological conditions are required [34]. This type of reaction is based on the reaction between thiolate anion and thioester to produce other thiolates and thioester products (Fig. 2a). Moreover, carboxylic acids are formed due to the hydrolysis of thioesters during the competing process in water. The exchange speed and hydrolysis speed depend on the temperature and pH value of solution, as well as the acidity of the thiol-variant. For the fixed physiological environment, the rate-determining process during this type of reaction is decided by the relative pK_a_ of entering and leaving thiols [35]. When the pK_a_ of the conjugate acid in nucleophilic thiolate is greater than leaving thiolate, the rate-determining process occurs in the production of tetrahedral intermediate. Oppositely, when the pK_a_ of conjugate acid in attacking thiolate is less than leaving thiolate, the rate-determining process occurs in the disintegration of tetrahedral intermediate. Notably, the reaction between thioester and nucleophilic oxygen is slower than the reaction between thioester and nucleophilic sulfur, which favors the production of thiol-thioester exchange products rather than hydrolysis [35].

Grinstaff et al. studied dissolvable hydrogels made by thiol-thioester exchange reaction [36, 37]. In particular, they studied a hydrophilic poly(ethylene glycol) lysine sulfhydryl (PEG-LysSH) dissolvable hydrogel-made stem from thiol-thioester exchange (Fig. 2b). To prepare the PEG-LysSH, the PEG amine (*M*_w:_ 2 kDa) was introduced on the lysine-based peptide dendron possessing four terminal thiols by a standard peptide coupling reaction. The dendritic molecules were composed of repeating nanoscale motifs, which were somewhere between polymers and a small organic molecule systems. Dendrons formed easily via a variety of non-covalent interactions, and they could provide a macromer with many reactive sites to ensure the fast formation of hydrogels [38]. The use of a dendritic macromonomer provides many advantages such as fine control of the composition, structure, and molecular weight. Then, the dissolvable thioester-linked PEG-LysSH hydrogel was fabricated spontaneously within seconds by mixing poly(ethylene glycol disuccinimidylvalerate) (SVA-PEG-SVA) with dendritic macromonomer with multiple reactive positions via native chemical ligation (NCL) reaction [39, 40]. The dissolution of PEG-LysSH-based hydrogel could occur in both l-cysteine methyl ester (CME) and 2-mercaptoethanesulfonate (MES) solutions. The dissolving mechanism of the hydrogel depended on thiol-thioester exchange reaction between the thioester bonds in hydrogel and thiolate solutions (e.g., CME, MES), and formed amide linkage to preclude re-formation of hydrogel. They found that the concentration of thiolate solutions and pH values had a significant effect on the dissolution behavior of PEG-LysSH hydrogel. The thioester bridges in the hydrogel could rapidly cleave, and the gel completely dissolved within 24 min at pH 8.5 in 0.3 M of MES solution. Since the hydrogel could be easily removed from the skin to avoid secondary damage, in vivo experiments had shown that this type of hydrogel possesses a potential application in wound repair. Recently, Konieczynska et al. synthesized a stimulus dissolvable dendritic thioester hydrogel burn dressing for second-degree burn healing [41]. The hydrogel was composed of a lysine-based dendron and a PEG-based cross-linker. Moreover, the lysine-based dendron used in the hydrogel was capped with nucleophilic amines, which could overcome the limited storage life and fine controllable gel rate that allows the matrix to easily fill the complex geometry of the burned area. They further studied the performance of on-demand dissolution of this hydrogel. After the hydrogel was applied to a second-degree burn wound on a rat and left to gel for 1 h, a CME-soaked gauze was administered to half of the hydrogel for 30 min, resulting in the dissolution of the gauze-treated hydrogel.

Overall, these works suggest that the on-demand dissolution of hydrogel based on thiol-thioester exchange provides a relatively inexpensive (Sigma Aldrich, 25 g of CME for $28.60) and desirable alternative to debridement of the dressing. However, the thiols as hydrogel precursors are easily oxidized to disulfides, which results in the disactivation of the moiety in formation of hydrogel. Moreover, the toxicity of thiolate solution needs to be considered, and the dissolving and adhesive behavior of hydrogels in highly wet wound environment with high hydraulic pressure should be further studied. Herein, we summarize the main features, advantages, and disadvantages of these hydrogels in Table 1.

**Table 1:** Some of the main features of various crosslinking types of hydrogels. *PBS* phosphate buffer saline

	Classification	Exogenous dissolution agents	*In situ* formation or not	Expected dissolution time	Potential for wound treatment	Advantages	Disadvantages
Chemically cross-linked hydrogels	Stimuli-sensitive hydrogels	Nothing	Yes	Immediately	Better application *in vivo*	Hydrogel are pure and less toxic	Low mechanical strength, less crosslinking species, less selectivity of polymer, long gelation time
	Supramolecular self-assembly hydrogels	Mild chemical irrigant	Yes	Within 2 min	Better application *in vivo*	Hydrogels have better mechanical properties and less toxic effects	Self-assembly process is difficult to control
Physically cross-linked hydrogels	Thiol-thioester exchange	Thiolate	Yes	Within 25 min	Better application *in vivo*	A cheaper way for hydrogel dissolution	Dissolution times of hydrogel are too long, and toxicity of thiolate is unknown
	Thiol-disulfide exchange	Thiol-containing reducing agent	Yes	Within 10 min	Better application *in vivo*	Built-in redox-sensitivity as living cells	Cytotoxicity of hydrogels or dissolution agents are uncertainty
	Retro-Michael reaction	Glutamate, PBS (pH 7.4), or light	Yes	2 days (glutamate); 4 days (PBS) 4.5 min (light)	Further research is needed	Increased stability for sustained release under highly reducing conditions	Michael acceptors for retro Michael Reaction have been less studied, and the effect of hydrogel dissolution is poor with side reaction
	Retro-Diels-Alder reaction	Dimethy formamide	No	0.4 h (100 °C)	Further research is needed	Hydrogels are formed need no catalysts or initiators	The dissolution temperatures of hydrogel are too high

##### Thiol-disulfide exchange

**Fig. 3:**
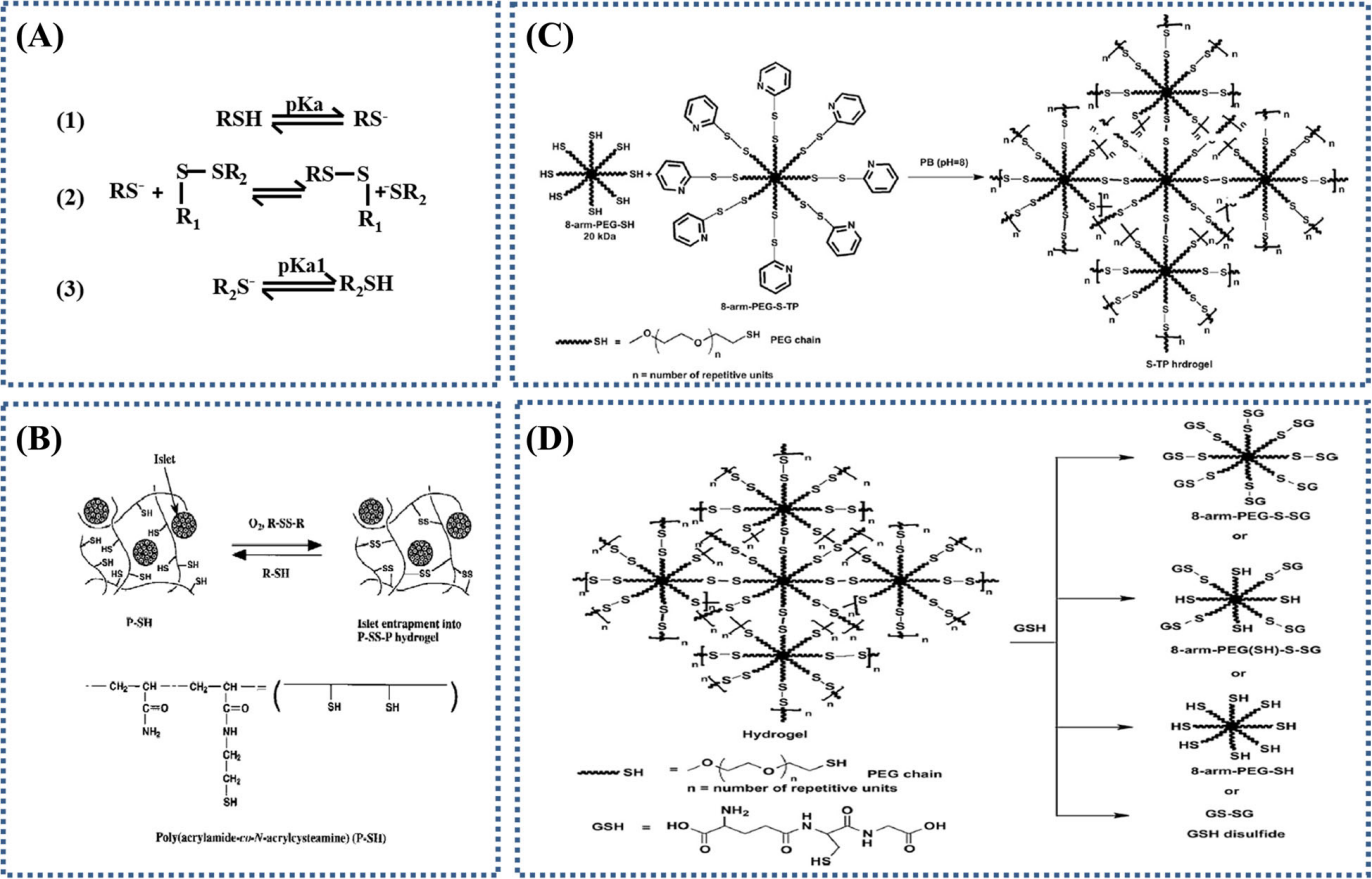
Thiol-disulfide exchange reaction based hydrogels formation and theirs dissolution. **a** Thiol-disulfide exchange reaction. Figure is adapted with permission from the original articles of Houk and Whitesides [43] (Copyright 1987 by Amerian Chemical Society). **b** Reaction scheme for hydrogel preparation and its reliquefaction. Figure is adapted with permission from the original articles of Hisano et al. [45] (Copyright 1988 by John Wiley & Sons, Inc.). **c** Schematic representation of thiopyridyl terminations appended on the 8-arm-poly(ethylene glycol) (PEG)-SH to form 8-arm-PEG-S-TP. Thiopyridine is a good leaving group and the 8-arm-PEG-S-TP forms disulfide bridges with the 8-arm-PEG-SH in phosphate buffer (PB) (pH 8) resulting in S-TP hydrogels [47]. **d** Schematic of the reversible nature of hydrogels. Glutathione (GSH) acts as a thiolate moiety and attacks the disulfide bonds resulting in the breakdown of the hydrogel network (gel to sol transition). The possible products are 8-arm-PEG-SH, 8-arm-PEG-(SH)-S-SG, 8-arm-PEG-S-SG and GS-SG. Figures are adapted with permission from the original articles of Anumolu et al. [47].(Copyright 2010 by Elsevier Ltd.)

The thiol-disulfide exchange reaction is important to many biological processes including the formation of cysteine disulfide bonds and disulfide mediated redox reactions [42, 43]. Typically, thiol-disulfide exchange contains three reversible steps (Fig. 3a): (1) ionization of thiol to thiolate anion in basic medium, (2) thiolate anion attack on the sulfur atom of the disulfide moiety via SN_2_ mechanism, and (3) protonation of thiolate anion. Due to its reversibility, the thiol-disulfide exchange has attracted interest in mediating changes in the modulus of hydrogels. The disulfide bond is a popular dynamic covalent bond that can respond to light stimulus and redox reagents [44]. Generally, the reversible thiol-disulfide exchange reaction can be triggered with excess thiolate and lofty pK_a_.

Early preparations of on-demand disolving hydrogel according to thiol-disulfide exchange reaction were described by Hisano et al. [45]. The soluble poly (acrylamide-co-*N*,*N*′-bisacrylcystamine) (P-S-S-P) hydrogel was formed through air oxidation of the thiols to disulfide bonds (24 h) or thiol-disulfide exchange reaction with poly(acrylamide-co-*N*-acryl-cysteamine) (P-SH) and low molecular weight disulfides (3,3′-dithiodipropionic acid, glutathione disulfide, or cystamine) [45]. l-cysteine or glutathione (GSH) molecules were used to dissolve this hydrogel via thiol-disulfide exchange reaction (Fig. 3b). The dissolution time of hydrogel reduced with the increase of the concentrations of l-cysteine or GSH, and the hydrogel could be dissolved within 1 min by adding the l-cysteine or GSH at a concentration of 600 μmol/mL. Recently, Szilagyi et al. developed a redox-responsive disulfide cross-linked polysuccinimide (PSI) gels, which showed a reversible dissolution and gelation performance based on thiol-disulfide exchange reaction in a shorter time [46]. The PSI gels were dissolved within 15 min with the reducing agent of dithiothreitol at 1 mM, and the regelation phenomenon occurred with the PSI gels were oxidized in air to disulfide linkages within 4–6 h. Anumolu et al. [47] designed analogous, and better hydrogels, which were composed of 8-arm-PEG-SH and either H_2_O_2_ or 8-arm-PEG-sulfur-thiopyridine (Fig. 3c). The hydrogels were in situ cross-linked in phosphate buffer saline (PBS) (pH 8.0) within 60 s and 10 s, respectively. GSH was added as the thiolate moiety to leave extant disulfide bonds (Fig. 3d) [47]. The hydrogels were dissolved within 30–40 min, 15–20 min, and 10–15 min in the presence of the 1%, 3%, and 5% (*w*/*v*) glutathione solutions, respectively.

All of these works provide ways to synthesize on-demand dissolvable hydrogels according to thiol-disulfide exchange with the goal of wound dressing application. Unfortunately, as shown in Table 1, there are little data to speak to the toxicity of the dissolution agents. One more problem is that the thiol-containing hydrogel precursors are easily oxidized in air, causing trouble for the stable synthesis of the hydrogels.

##### Retro-Michael reaction

**Fig. 4:**
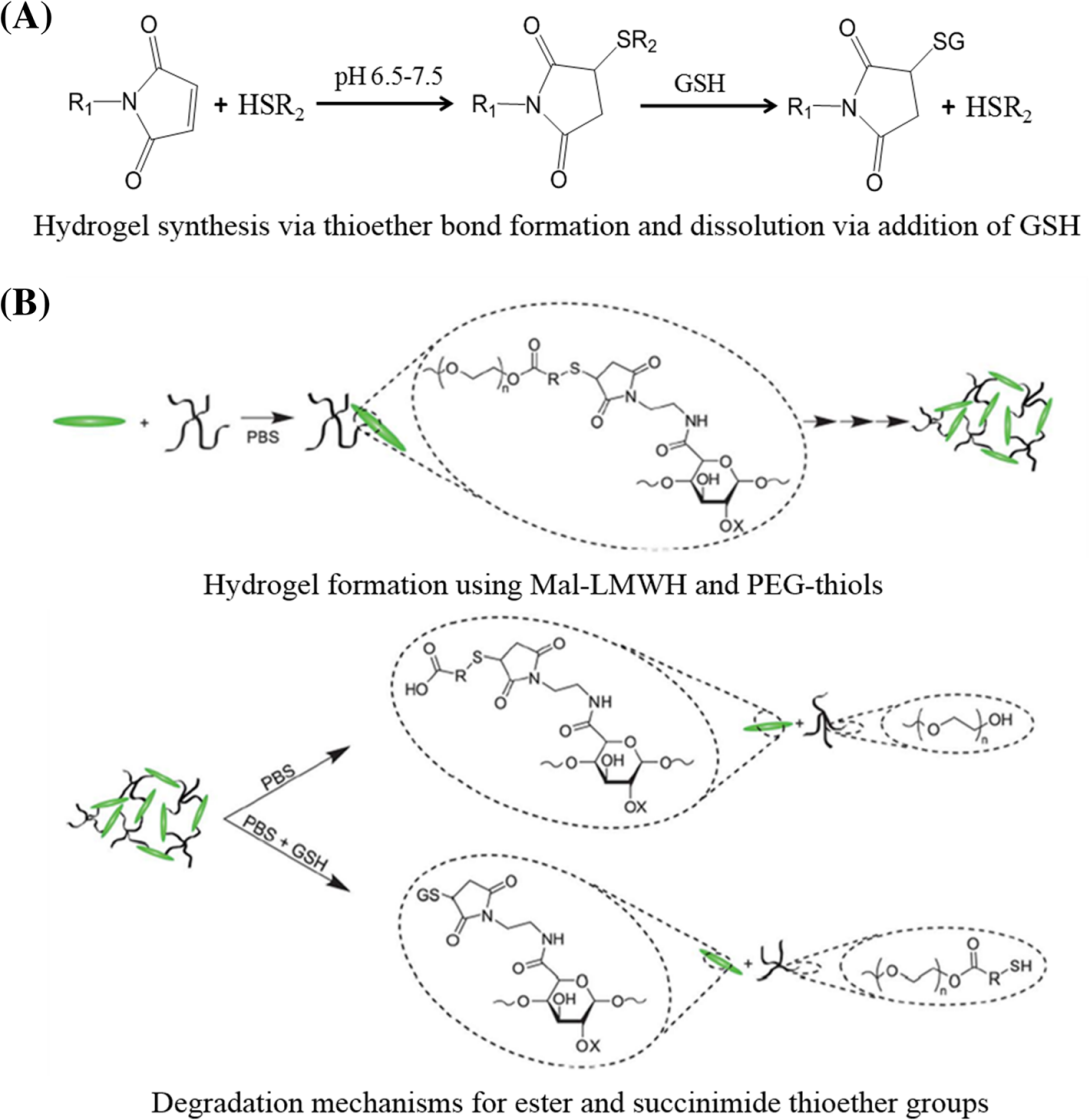
Formation and dissolution of hydrogel based on retro-Michael addition reaction. **a** Michael addition and retro-Michael reaction. Figure is adapted with permission from the original articles of Konieczynska and Grinstaff [36] (Copyright 2017 by American Chemical Society). **b** Hydrogel formation using maleimide-functionalized low-molecular weight heparin (MAL-LMWH) and poly(ethylene glycol) (PEG)-thiols; degradation mechanisms for ester and succinimide thioether groups. Figure is adapted with permission from the original articles of Baldwin and Kiick [48] (Copyright 2013 by Royal Society of Chemistry). *GSH* glutathione, *PBS* phosphate buffer saline

The on-demand dissolution hydrogels can also be fabricated by retro-Michael reaction. One of the common retro-Michael reaction-based hydrogels use maleimide (MAL)-functionalized macromolecular monomers crosslinking with many thiol-functionalized multi-arm polymers to form a network crosslinking with thioether connections. The dissolution mechanism of the retro-Michael reaction-based hydrogels consists of covalent bond shift from original succinimide thioether compounds to jarless GSH conjugate with substantial reducing agents (Fig. 4a). The rate and degree of the dissolution reaction are regulated though the retro-addition rate, which are controlled via modulating the activity of the Michael donor. Typically, a Michael donor with lofty pK_a_ can be used to dissolve the hydrogel rapidly in reductive condition, while the retro reaction will be blocked in the case of the Michael donor with a high enough pK_a_.

One example of the dissolvable hydrogels based on succinimide-thioether cross-linking was formed in situ by using a mixture of thiolated 4-arm-PEG (*M*_w_: 10 kDa) and MAL-functionalized low-molecular weight heparin (MAL-LMWH) (Fig. 4b) [48]. With the addition of GSH, the hydrogels were dissolved, and the dissolution rate was governed by the reducing conditions [48]. In another recent report, Kiick et al. investigated an on-demand dissolvable hydrogel, which could be fabricated in situ within seconds via a Michael-type addition reaction [49]. The hydrogel was then dissolved though three different modes: GSH-reducing environment, photocleavage (visible and two-photon infrared light), and ester hydrolysis [49, 50]. Compared with disulfide-based hydrogels, these succinimide-thioether bond containing hydrogels exhibit higher stability. Current reports on the preparation of dissolvable hydrogels based on retro-Michael reaction focus on precursors containing maleimide, and more Michael acceptors (e.g., acrylates) for preparation of these hydrogels also need to be studied.

##### Retro-Diels-Alder reaction

**Fig. 5:**
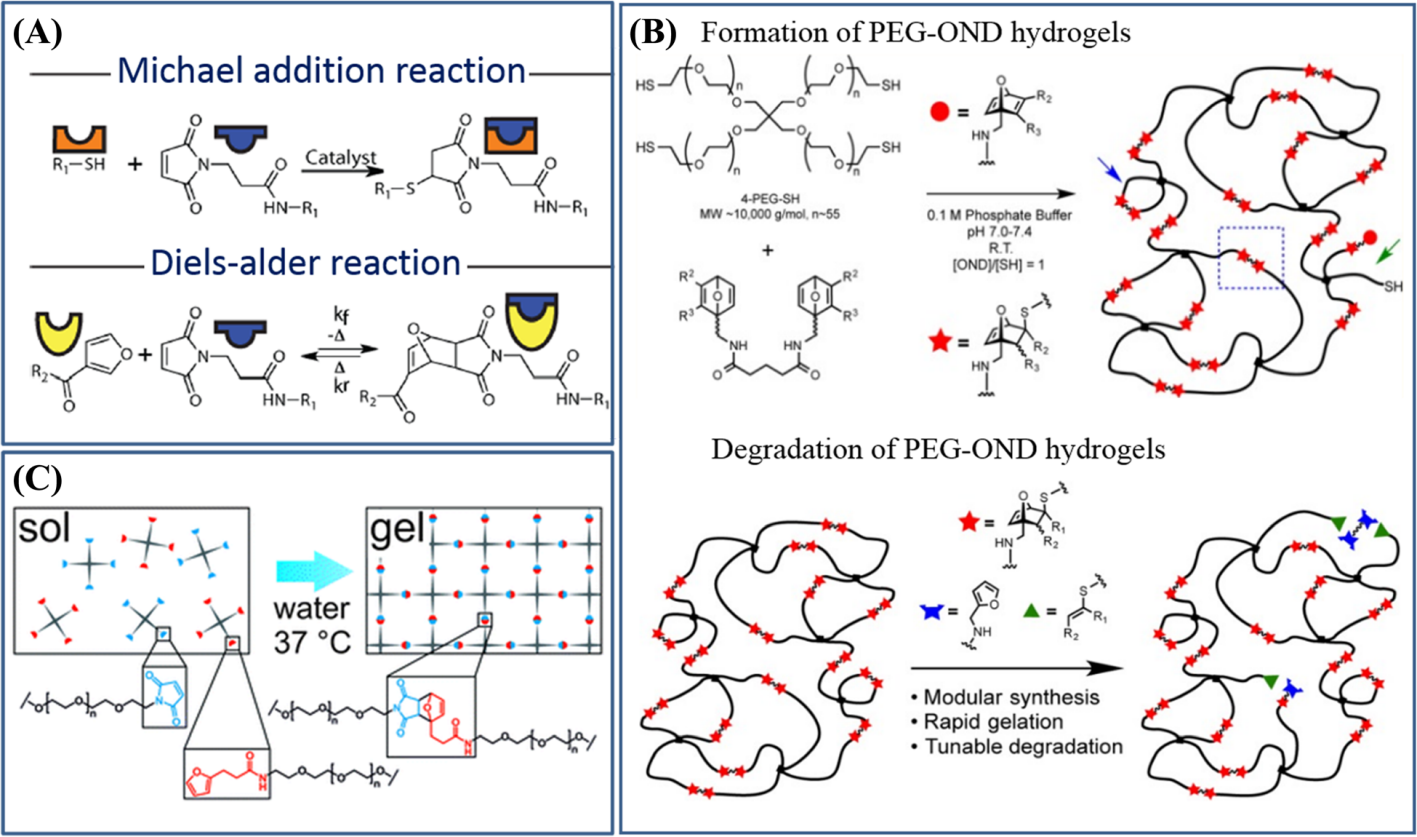
Retro-Diels-Alder reaction based hydrogels formation and their dissolution. **a** Michael addition and Diels-Alder (DA) reaction. Figure is adapted with permission from the original articles of Koehler et al. [51] (Copyright 2013 by American Chemical Society). **b** Formation and degradation of poly(ethylene glycaol)-oxanorbornadiene (PEG-OND) hydrogels. Figure is adapted with permission from the original articles of Higginson et al. [53] (Copyright 2015 by American Chemical Society). **c** The DA reaction was investigated as a cross-linking mechanism for PEG-based hydrogels. Figure is adapted with permission from the original articles of Kirchhof et al. [54] (Copyright 2013 by Royal Society of Chemistry)

Another type of on-demand dissolvable hydrogels is based on Diels-Alder (DA) reactions and rDA reactions, and these reactions are reversible by temperature (Fig. 5a) [51]. Generally, DA reaction-based hydrogels are prepared in a reaction medium of water by the highly specific cyclization reaction of substituted alkene dienophile with conjugated diene. The reversible reaction-based hydrogels require high temperature to break the bond and dissolve. Moreover, the dissolution of these hydrogels requires many rDA reactions to happen synchronously at a fast rate. These effects limit their translation into biological milieus. Thus, there is interest in a new type of hydrogel systems with high reactivity to overcome the drawbacks above.

Wei et al. reported a dissolvable hydrogel that could be prepared via DA cyclization, and it could be dissolved at above 70 °C via rDA reactions by exposing it to dimethylformamide (DMF) [52]. In order to make this class of functional hydrogels apply in a physiologically relevant environment, Finn et al. prepared the PEG-oxanorbornadiene (OND) hydrogels that could be dissolved in more than 1 day in a biologically relevant environment via rDA-mediated reactions (Fig. 5b) [53]. The hydrogel was synthesized via the reaction between 4-arm thiol-terminated PEG (*M*_w_: 10 kDa) and 7-OND cross-linkers. Interestingly, the authors observed that its dissolution rate was related to the temperature and OND moiety without swelling buffer, which was not related to pH values (5.0–9.0) during swelling process. Another related study of the dissolvable hydrogel with DA reaction was reported by Kirchhof et al. [54]. The DA hydrogel was prepared by mixing equimolar amounts of furyl and MAL substituted multi-arm PEGs [54] (Fig. 5c). Moreover, the gelation time and mechanical properties of the DA hydrogel were related to the concentration of polymer, branching amount, and molecular weight of PEGs [54]. The dissolution of hydrogel was triggered by the hydrolysis of chemically inert meleamic acid derivatives, and the process usually needs days to weeks [54].

Although there are other reversible open-loop addition reactions that are similar to the DA reaction and can be used to prepare dissolvable hydrogels, most can only be dissolved under radiation of the ultraviolet light (100–315 nm), which is harmful to humans [55]. As shown in Table 1, the DA/rDA transformations provide an efficient, economical, and simple method for the formation and dissolution of hydrogels (e.g., the dissolution of hydrogels does not require any exogenous agents at elevated temperature). The slow dissolution of these hydrogels at biologically relevant conditions severely limits their biological applications.

#### Physically cross-linked hydrogels

**Fig. 6:**
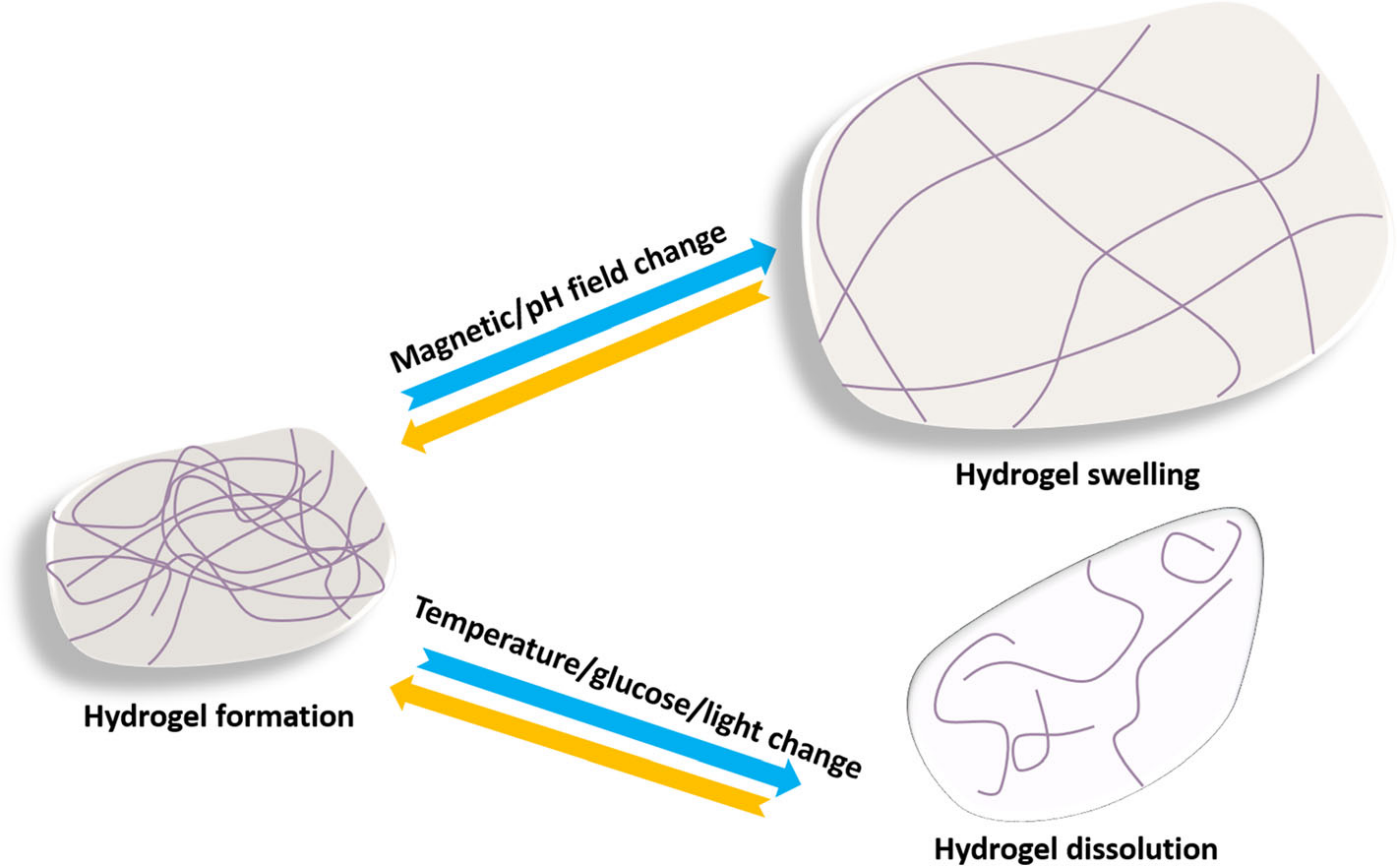
Schematic illustration of the dissolution or swelling behavior of stimuli sensitive physically cross-linked hydrogels

The formation of physically cross-linked hydrogels is based on the physical interactions (e.g., ionic and hydrophobic associations) without any toxic cross-linking agents [56, 57]. This type of hydrogel can be reversed by the physical interactions between the molecular chains, and such hydrogels are typically safe in clinical applications [58]. Some physically cross-linked hydrogels can undergo volume or gel-sol phase reversible transitions in response to environmental stimuli, as shown in Fig. 6 [59]. Hydrogels containing such “sensor” properties have vast application prospects in biomedical fields because they can be removed atraumatically from wounds and because they are biodegradable. In general, the phase transition from gel to sol of physically cross-linked hydrogels is a slow process (which is usually called dissolution process). At present, different dissolution mechanisms have been proposed to explain the dissolution of all kinds of polymers, and the process can be roughly divided into two stages of swelling and dissolution [60, 61]. The dissolution of crystalline or semicrystalline hydrogels is accompanied by a decrease in the degree of crystallinity until disappearing [62]. The time required for complete dissolution is related to the molecular weight, crystallinity, and other factors of hydrogels, and the time ranges from several seconds to several weeks. However, the clinical application of traditional physically cross-linked hydrogels still faces the problem of uncontrollable dissolving and poor mechanical properties [63]. What is exciting is that the invention of supramolecular hydrogels in recent years may overcome these shortcomings [64]. Supramolecular hydrogels are also often sensitive to the environment, and the dissolution of these hydrogels via stimulus is an ideal method for preventing secondary injury in wound care [65, 66]. The recent progress of stimuli-dissolving traditional physically cross-linked hydrogels and supramolecular hydrogels are summarized in this section.

##### Temperature-sensitive physically cross-linked hydrogels (TPCH)

Many environmental stimuli have been used to induce the volume or phase reversible transitions of the hydrogel systems, including pH, temperature, ions, electric fields, light, pressure, sound, and magnetic fields [67, 68]. Recently, the gel-sol phase reversible transitions of temperature-sensitive physically cross-linked hydrogels have been widely studied [69, 70]. Here, the TPCH are mainly introduced.

The temperature-sensitive behavior of physically cross-linked traditional hydrogels means that they can change their hydrophilicity, hydrophobicity, and volume of gel networks. They can also undergo gel-sol reversible transitions with the change of temperature [71]. The polymer chains in TPCH can undergo sol-gel phase reversible transitions, which are sol at low temperature and gel at high temperature [72]. Typical TPCH are composed of hydrophobic chains (e.g., poly(*N*-isopropylacrylamide)) and hydrophilic links (e.g., poly(tetramethyleneether glycol)), and their molecular architecture may include two-block, three-block, multi-block, and hyperbranched structures [73]. The polymers can form a semi-rigid gel through the hydrophobic interactions or secondary bonding, and these bonds between the polymer chains can be changed with increased temperature [74]. TPCH can undergo the hydrophilic-hydrophobic transition via the hydrophobic interaction when temperature is above the lower critical solution temperature (LCST) [75]. The polymer solution has the low viscosity at room temperature, but it will turn into gel when the temperature is above the LCST. LCST values can be adjusted by changing the ratio of hydrophobic chain, hydrophilic chain, and molecular weight [76]. Generally, TPCH are significant for wound care because they can be removed easily from the wound at a temperature below human physiological temperature (37 °C).

One example of TPCH preparation is a physical mixture of chitosan and glycerol phosphate (GP) disodium salt [77]. The mixture remains in a transparent liquid state at room temperature, while gelation occurs at 37 °C. The gelation occurs because phosphates in GP neutralizes the amine groups of chitosan, leading to a rise in hydrophobic and hydrogen bonding between chitosan chains at a high temperature. Bhattarai et al. introduced an injectable chitosan-PEG (45–55 wt%) TPCH, which took advantages of the interactions among chitosan chains for gelation [68]. The chitosan-PEG co-polymer was synthesized by chemically grafting monohydroxy PEG onto chitosan backbone via Schiff base and sodium cyanoborohydride chemistry. The mixture could be injected using a 22-G needle below the transition temperature, and gelation occurred at approximately 25 °C. The hydrogen bonds between PEG and water molecules are dominant at low temperatures, while the hydrophobic interactions between the polymer chains are dominant at high temperatures [78, 79], leading to the formation of hydrogels by the hydrophilic-hydrophobic transition. As wound dressing, this temperature-sensitive hydrogels can be dissolved on demand just by changing the temperature. Moreover, chitosan is able to promote faster wound healing and generate smooth scarring because of the enhancement of vascularization and the supply of chitooligomers at the lesion site [80, 81].

Such polymers can be used as an injectable hydrogels for treating the irregularly shaped wound; they can be injected around the wound at low temperatures, forming a gel at human physiological temperature. They can also be used as an in-situ gel sealant for emergency wound treatment, which can rapid gelation to attaching to the wound and be removed easily before subsequent treatment. However, as is shown in Table 1, one of the big problems is that the gelation time is rather long. Moreover, the dissolved temperature of TPCH should be further optimized.

##### Other stimuli sensitive physically cross-linked hydrogels

In addition to the wide application of TPCH discussed above, other stimuli have also been used for preparing dissolvable hydrogels with environmental sensitivity. However, the preparation of hydrogels purely on the basis of physical cross-linking is rare, and the variety of polymers is limited. Nevertheless, there are several other stimuli-sensitive physically cross-linked dissolving hydrogels that have been studied. For example, the pH values can affect swelling or shrinkage behavior of pH-sensitive hydrogels, because the pendant acidic (e.g., carboxylic acids) or basic (e.g., ammonium salts) groups in solution can accept or release protons with the change of pH values [72, 82]. These kinds of hydrogels have been most widely used in the field of controlled drug delivery and permeation switches [82]. Glucose-sensitive hydrogels can undergo sol-gel phase reversible transitions depending on the glucose concentration in the environment [72]. Some glucose-sensitive hydrogels can be formed by a reversible crosslink among the glucose-containing polymer chains via the non-covalent interaction between concanavalin A (Con A) and glucose. The glucose binding sites in Con A can combine free glucose or polymer-bound glucose depending on the concentration of free glucose [83]. Magnetic-sensitive hydrogels may undergo phase transitions via a magnetic field, and one way to obtain such hydrogels is to add magnetic nanoparticles [84]. Light-sensitive hydrogels are usually synthesized by introducing photo-responsive groups (e.g., azobenzene) [85, 86]. The self-assembly structure of the light-sensitive hydrogels can be destroyed with molecular isomerization upon ultraviolet irradiation; therefore, most of these hydrogels can undergo sol-gel phase transitions in the response process [87].

##### Supramolecular self-assembly hydrogels

**Fig. 7:**
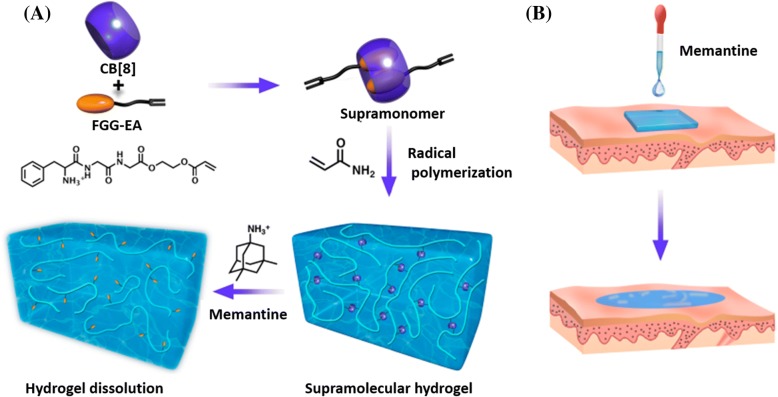
Schematic depiction of **a** supramolecular hydrogel fabrication from supramonomers and its dissolution process upon memantine irrigation [96] and **b** its application as wound dressing materials. Figures are adapted with permission from the original articles of Xu et al. [96] (Copyright 2017 by American Chemical Society). *CB* cucurbit, *FGG-EA* Phe-Gly-Gly ester derivative

Supramolecular hydrogels are developed by employing noncovalent interactions (e.g., electronic, host-guest, and hydrophobic interactions) between components [88]. Supramolecular hydrogels often feature reversible, adaptive, stimuli-responsive, self-healing, and degradable properties due to the dynamic nature of noncovalent interactions [89]. Typically, supramonomers are bifunctional monomers that are prepared by noncovalent synthesis, but they also can undergo traditional covalent polymerization [90]. Supramonomers can also be used as cross-linkers to fabricate supramolecular hydrogels that respond to stimulus (i.e., temperature, light, pH, electric field and oxidation reduction) and degradable properties. Since its unique dynamic and degradable properties, supramolecular hydrogels have been used as wound dressings that will be capable of dissolution according to the demand. Recently, researchers have been working on the stimuli sensitivity of the gel-sol phase reversible transitions of supramolecular hydrogels. For example, poly(*N*-isopropyl acrylamide) (PNIPAM) is often used to prepare temperature-sensitive supramolecular hydrogels. This system features hydrophilic amide group and hydrophobic isopropyl groups [91]. In addition, the supramolecular hydrogel [92], which is connected by β-cyclodextrin (β-CD) and PEG, has an excellent temperature sensitivity. The transition can be repeated multiple times without significant modulus loss. In recent years, the researches on ultraviolet-visible-sensitive supramolecular hydrogels have received wide attention [93, 94]. The most typical example consists of host molecule (e.g., cyclodextrin) and guest molecule (i.e., azobenzene and its derivatives). Such a hydrogel can undergo gel-sol phase reversible transitions with the irradiation of ultraviolet or visible light. Azobenzene can isomerize under ultraviolet or visible light, which will cause changes in the whole hydrogel system [95]. The host-guest interactions between the tripeptide (Phe-Gly-Gly) ester derivative and cucurbit [8] uril (CB[8]) were employed to synthesize supramolecular cross-linkers with one acrylate moiety at each end [96]. Then, supramolecular hydrogels were fabricated by copolymerization of acrylamide (AAm) with the above supramolecular cross-linkers (Fig. 7) [96]. Similar to the traditional chemically cross-linked hydrogels, the supramolecular hydrogels are biocompatible, soft, elastic, water-absorbant, and capable of being loaded with therapeutic agents. Moreover, due to the fact that these hydrogels are composed of dynamic and reversible supramolecular cross-linkers, the supramolecular hydrogels will dissolve quickly (within 2 min) with memantine. As a result, the stimuli-dissolving supramolecular hydrogels will present a new generation of wound dressing materials.

Supramolecular hydrogels have been widely researched as wound dressing due to the advantages of degradable, injectable, adjustable gelation process, simple preparation, and without chemical reactions. More important, this self-assembled supramolecular hydrogels as wound dressing can be mildly removed after finishing its work. However, it is difficult to control self-assembly process. The optimization of the amphiphilic polymers of building blocks provides a possible for controlled self-assembly process, while the gelation time and dissolved response speed need to be further improved.

## Conclusions

In this paper, four popular strategies for preparation of dissolvable chemically cross-linked hydrogels, environment-sensitive physically cross-linked hydrogels and supramolecular self-assembly hydrogels are introduced. All of them provide economical and effective methods for the synthesis of controlled and on-demand dissolving hydrogels for *in vivo* applications.

It should be pointed out that different types of wounds and different stages of the same wound have different requirements for hydrogel dressings, and it is difficult for a single material to meet the complex needs of the wound. Therefore, multifunctional dressings, which can be prepared by combining different functional materials, open up a method to meet the various demands in the healing process. Besides, new cross-linking and dissolution strategies should be considered in designing hydrogels for clinical purposes. Continued study and development of hydrogels is of great interest. Overall, the exploration of new type of on-demand dissolvable hydrogels is an area that displays the creativity of both chemists and biologist in materials and chemical biology.
